# Bacteria spatial tracking in Urban Park soils with MALDI-TOF Mass Spectrometry and Specific PCR

**DOI:** 10.1186/s13040-022-00318-6

**Published:** 2023-01-14

**Authors:** Diego Arnal, Celeste Moya, Luigi Filippelli, Jaume Segura-Garcia, Sergi Maicas

**Affiliations:** 1 Department of Microbiology and Ecology, Faculty of Biological Sciences (Universitat de València), 46100 Burjassot, Spain; 2 Department of Computer Science, School of Engineering - Universitat de València, 46100 Burjassot, Spain

**Keywords:** Enterobacteria, MALDI-TOF MS, 16S rDNA, Kriging

## Abstract

Urban parks constitute one of the main leisure areas, especially for the most vulnerable people in our society, children, and the elderly. Contact with soils can pose a health risk. Microbiological testing is a key aspect in determining whether they are suitable for public use. The aim of this work is to map the spatial distribution of potential dangerous *Enterobacteria* but also bioremediation useful (lipase producers) isolates from soils in an urban park in the area of Valencia (Spain). To this end, our team has collected 25 samples of soil and isolated 500 microorganisms, using a mobile application to collect information of the soil samples (i.e. soil features, temperature, humidity, etc.) with geolocation. A combined protocol including matrix-assisted laser desorption/ionization time of flight mass spectrometry (MALDI-TOF MS) and 16S rDNA sequencing PCR has been established to characterize the isolates. The results have been processed using spatial statistical techniques (using Kriging method), taking into account the number of isolated strains, also proving the reactivity against standard pathogenic bacterial strains (*Escherichia coli*, *Bacillus cereus*, *Salmonella*, *Pseudomonas* and *Staphylococcus aureus*), and have increased the number of samples (to 896 samples) by interpolating spatially each parameter with this statistical method. The combined use of methods from biology and computer science allows the quality of the soil in urban parks to be predicted in an agile way, which can generate confidence in its use by citizens.

**Supplementary Information:**

The online version contains supplementary material available at 10.1186/s13040-022-00318-6.

## Introduction

Urban population is growing in most countries around the world. Almost 79% of the Spanish population inhabits urban areas with more than 40,000 people [1]. Increasing urbanization is leading to higher population density in cities, which in turn threatens sustainable development and creates many environmental and social problems. Human beings are in continuous contact with soil, directly or via food, air or water [2]. The contact with soil in gardens is even more frequent for infants and elder people. Many of the developing countries are facing more problems of higher metropolitan agglomerations as the pace of urbanization is faster in developing nations which is gravely surpassing the capacities of these cities to provide adequate facilities to the urban inhabitants [3, 4]. In addition to the higher population densities in the developing areas, the economic growth and modern life style of urban inhabitants is the cause of serious contamination of soil and water ecosystems [5, 6].

In contrast, urban soils are considered a fundamental ecological asset for cities and land-use planning [7, 8]. These soils deserve more attention, than ever, due to substantial increase in interests of urban dwellers to use them for urban agriculture, gardening, and landscaping. The sustainable management of soil and water resources is very critical in urban environment, particularly, in the developing countries [6]. A very crucial issue in the sprawling urban dwellings is the handling and management of municipal wastes and sewage water [6, 9]. Soil is a common recipient of solid wastes able to contain enteric bacteria, which may be potentially pathogenic and facilitate the transmission of enteric diseases. Gastroenteric diseases are the most common infections and generally associated to species such as *Salmonella* sp., *Vibrio* sp. or *Escherichia coli* [10]. Soil-associated diseases are caused by opportunistic pathogens that belong to the normal soil microbiota or come from enteric pathogens which are present in soil via human or animal excreta. The entry of important amounts of enteric pathogens into the soil environment are the use of animal excreta as manure and the improper disposal of human excreta in gardens. Moreover antimicrobial resistance examples in urban areas have been reported [11]. Antibiotic resistant bacteria with resistance phenotypes and genotypes are ubiquitous in the parks and have become a global health concern [12]. A high prevalence of antibiotic-resistant bacteria in the environment, in direct contact with humans, is possibly one of the most important threats to public health today [13]. Excessive use, and misuse of antibiotics have inevitably increased the environmental concentration of antibiotic resistance among bacteria, especially among enterobacteria [14, 15]. The richness of bacteria in urban parks may exacerbate the problem of resistance [16].

Wastewater can also have many contaminants while others may not be aware of the fact that untreated wastewater is a good habitat for many human pathogens [6, 17]. Nonetheless, numerous reports in literature reveal that the untreated municipal wastewater contains pathogenic microorganisms, which if transfered to the surface and groundwater bodies could result in the outbreak of diseases [18].

In the present research work an integrated biological and statistical study of bacterial communities in a defined urban park has been developed. We have combined basic microbiological techniques with modern identification tools and the spatial distribution of potential antibiotic producing isolates from soils. The results have been processed using spatial statistic techniques (mainly Kriging), taking into account the number of total isolated strains, enterobacteria and fungi. Lipase production was also assayed.

## Materials and Methods

### Study area

This study was carried out in the urban community park *La Granja* in Burjassot, València, Spain during 2019-20. The garden used municipal and rain water for irrigation which is a common practice in many urban community gardens in metropolitan València. A preliminary survey of the area was carried out by computer and visual inspection to decide which points could be most representative, based on their frequent use by man and animals. The action of both is expected to have a major impact on soil characteristics. Sampling was carried out from 25 different points, at a depth of 0-5 cm. Samples were put into a screw-tub and transported to the laboratory for bacterial isolation. Remaining samples were stored at -20$^{\circ }$C for further analysis.

### Soil characterization

#### pH measurement

Soil pH was measured by dissolving 1 g of soil in 5 mL distilled water, shaking for 2 min and then waiting 30 min for the soil to settle (assays by triplicate using a pH-meter Consort). A similar procedure was used with pH papers, dissolving 0.5 g of soil in 1 ml distilled water, vortexed for 5 min, and applied in small amounts to pH indicator strips (Universal Test Paper). The pH strip accuracy was tested against standard pH calibration solutions.

#### Color, texture and carbonate content determination

Soil color was determined by visual inspection and comparison of the samples against a Munsell soil standard chart [19]. Soil texture characteristics were used to classify soils according to the size of the different particles that compose it. The determination of the presence of carbonates in the soil was performed in a reaction that gives rise to effervescence, adding a few drops of 1:1 HCl to the soil samples. The more intense the effervescence, the higher the calcium carbonate content in the soil.

### Microbial isolation

A total of 1 g of soil was suspended in 10 mL of sterile water. Afterwards, serial dilutions were prepared and plated on 0.1 mL of the 1:100 to 1:100,000 dilutions in trypticase soy agar (TSA), MacConkey agar (MKA) and malt extract agar (MA) purchased from Conda (Madrid, Spain). After 48-72  h of cultivation, the total number of cultivable microorganisms were counted in the appropriate dilution plate of each culture media. Single colony was selected based on the morphology, color and size of bacteria and further purified three times on TSA broth agar medium by the repeated plate streaking method.

For the lipase assay, a total of 20 colonies from each TSA series were picked onto a new mother plate with the help of a grid. For the analysis of enterobacteriae, isolates from the MKA plates were grouped in different categories according to physiological and morphological criteria.

### Determination of Gram

Gram method for staining was used to classify the bacterial isolates from the TSA plates and determinate their microscopical morphology [20]. The preparations were observed in a clear field in a Microscope Eclipse E600 (Nikon) with a digital camera DS-Ri1 (Nikon).

### Determination of lipase activity

The 500 isolated bacteria were spread in four replicated Petri plates containing Tween-80 agar using sterile toothpicks. To demonstrate the production of extracellular esterases (lipases) the microorganisms were grown in this medium containing a synthetic lipid that presents ester links between sorbitol and oleic acid (Tween-80). It also contains calcium salts. If the microorganism has esterase activity (lipase), it will hydrolyze the ester link and release the oleic acid of the Tween-80. After 48-h incubation this oleic acid, in the presence of an excess of Ca$^{2+}$, will precipitate in the form of small crystalline oleate crystals that will form an opaque halo around growth.

### Microbial identification

#### 16 S RDNA partial sequence

At least one colony of different morphology was picked and refreshed in TSA plates. Bacteria DNA was extracted using a boiling method [21]. Primers SWI-F (5’ -AGAGTTTGATCCTGGCTCAG-3’) and SWI-R (5’ -GGTTACCTTGTTACGACTT-3’) were used to amplify the 16S rRNA gene. The amplification reaction was performed in a Primus 25 thermocycler (MWG, Ebersberg, Germany) under conditions previously described [22]. Direct sequencing of the PCR products was performed by ABBIPrism BigDye Terminator Cycle Sequence Reaady Reaction Kit (Applied Biosystems, Stafford, TX, USA) in the SCSIE service (Universitat de València (Spain). The sequences were aligned using the BLAST program, with complete sequences of 16S rDNA gene sequences retrieved from the EMBL nucleotide sequence data libraries [23].

#### MALDI-TOF

The identification of bacterial strains has also been carried out following the protocol recommended by Bruker Daltonics (http://www.bdal.de) by means of the extended direct transfer method. The strains were analyzed from fresh culture. The MALDI-TOF MS technique was performed using a Microflex L20 mass spectrometer (Bruker Daltonics) equipped with an N2 laser. All spectra were acquired in positive linear ion mode. The acceleration voltage was 20 kV [24]. The spectra were acquired as the sum of 240 shots per target. The mass range used for the analysis was 2,000-20,000 Da. Three spectra were obtained per strain by the MALDI Biotyper Realtime Classification (RTC) method. The resulting identification in front of the database MBT 7854 and MBT 7311_RUO (Bruker Daltonics), corresponds to the profile of the highest log score.

### Spatial statistical technique (Kriging method)

Spatial statistics allows the analysis of geolocated information by applying different methods, including Inverse Distance Weighting (IDW), spline interpolation, and Kriging. For this work we have used the Kriging approach, which is based on spatial autocorrelation [25]. The determined information from the samples of soil establish a data set in relation to different locations with its GPS coordinates, longitude and latitude, following the Kriging technique [26].

We have used RStudio as a framework for the R statistical programming language [27]. Within this framework, a bunch of libraries can be installed and used for the spatial statistical characterization of the region covered in this study (the script included explains the processing values) [22]. The parameters selected for the spatial statistical study, with Kriging method, were: pH, total amount of microorganisms (*Tufc*.*g*), amount of fungi (*Fufc*.*g*), amount of *Enterobacteria* ($T\_enterobac$), and finally, different groups of enterobacterial isolates which are related to lactose positive (Group I or *GI*), lactose negative (*GII*), slow fermentation (*GIII*), mucoid colonies (*GIV*) and other types (*GV*).

We have previously used the Kriging technique to evaluate some of these parameters in a wider area [22]. In the present work, we have used the “Matérn” model which involves partial-sill (PSill), Nugget, Range and kappa. This variogram is characterised by these parameters. The Sill is the value at which the model first flattens to a constant value representing the total variance where the empirical variogram appears to level off, and the Nugget is the value at which the semi-variogram nearly intercepts the y-value and is related to the amount of short-range variability in the data (here we will work with PSill which stands for “partial sill” and is the sill minus the nugget). The Range is the distance after which data are no longer correlated. Another useful parameter is kappa which measures the smoothness of the Matérn class of the variogram models.

The determined information from the samples of soil establishes a dataset in relation to different locations with their GPS coordinates, longitude and latitude. By denoting the determined value of the number of colonies or number of isolates at a specific location *x* as *Y*(*x*), this data set is defined as $\{Y(x),x \in D\}$, where *D* are all the locations of the modelling sets, following the Kriging technique. The objective of the proposed model is the forecast of $Y(x_o)$ in any location $x_o$, specifically those in the validation set. The measurement reports contain information about the set of covariables included. Therefore in Eq.1, *Y*(*x*) is modelled as an average of each covariable involved in the process in the geographical area considered plus some bounded spatial variability which is explained by the short-term process with spatial dependence, i.e.,

1 $$\begin{aligned} Y(x)=\mu (x)+\delta (x) \end{aligned}$$

where $\mu (x)=E[Y(x)]$ and $\delta (x)$ is a stationary Gaussian process with zero mean, whose spatial dependence characterization is given by the variogram $\gamma$ (Eq.2)

2 $$\begin{aligned} 2\gamma (h) = \textrm{Var}\left[ Y(x+h) - Y(x)\right] = \textrm{Var}\left[ \delta (x+h)-\delta (x)\right] , \end{aligned}$$

here *Var* denotes the variance and *h* is an offset.

## Results and Discussion

**Fig. 1:**
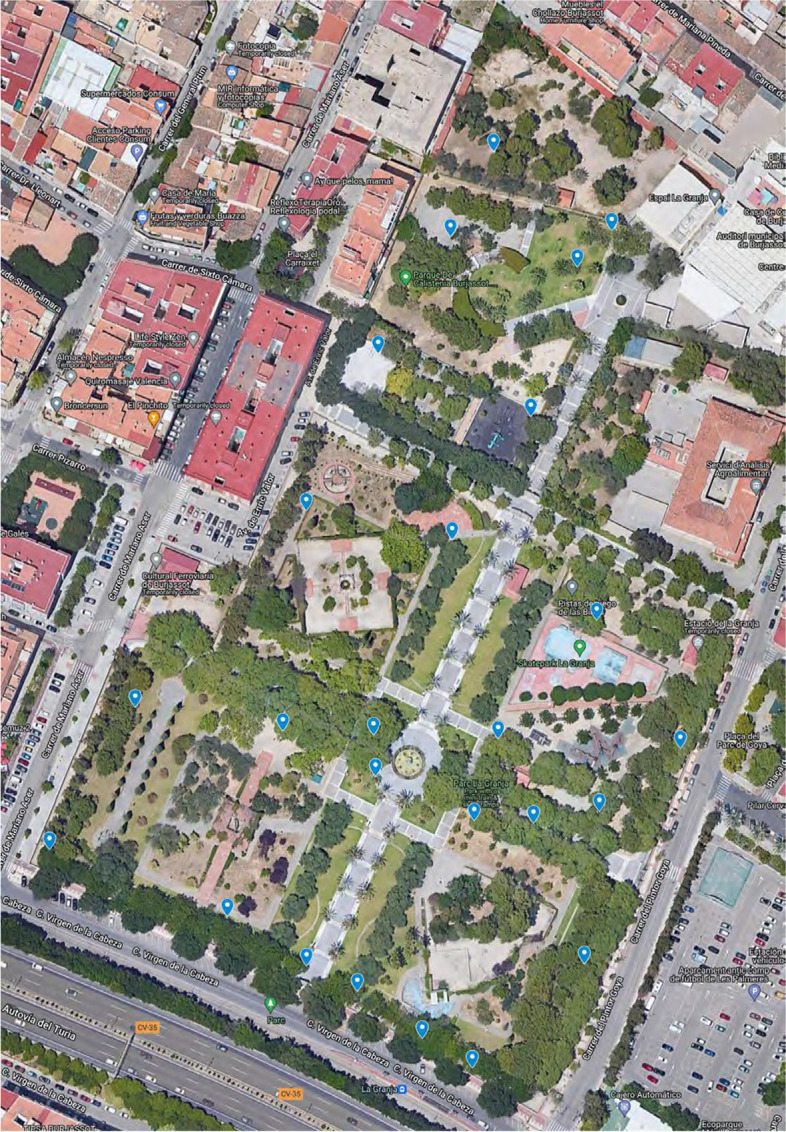
Park of La Granja. Geolocation of sampling points (Google Maps)

A total of 25 samples of soil were collected from the global area under study (La Granja Park, Burjassot, Spain) and processed as described in Materials and methods (Fig. 1). Geolocation and temperature (data not shown) were recorded in an Android based application previously reported [22].

### Characterization of physical and biological conditions of the area of study

We have carried out different tests to characterize soils. On the one hand, we have determined the color, one of the most significant properties, generally conditioned by the existence and proportion of organic and mineral compounds (Table 1). Most of the soils included in this study have a dark or brownish dark coloration. It is known that organic matter produces dark colors, usually blackish or brown. Therefore, it was expected to find a great quantity of organic compounds and, as a consequence, a high number of microorganisms (Table 1). The texture of soils is basically sandy all over the park, although some areas were described as sand silt or loamy sandy according to Lindbo et al. (2012) [28].

**Table 1:** Geolocation and basic soil characterization

Sample	Coordinates	pH	Color	Texture	Total m.o.)	Fungi )	Enterobacteria
code					(ufc$\cdot$g$^{-1}$)	(ufc$\cdot$g$^{-1}$)	(ufc$\cdot$g$^{-1}$)
GRA-01	39.504850, -0.414042	8.14	4/3	Sand Silt	7.0x10${^6}$	3.5x10${^4}$	6.4x10${^5}$
GRA-02	39.504623, -0.413252	7.73	3/3	Sand Silt	1.2x10${^7}$	1.0x10${^6}$	1.9x10${^6}$
GRA-03	39.504453, -0.412901	7.02	3/3	Sandy	6.3x10${^6}$	2.1x10${^6}$	3.3x10${^6}$
GRA-04	39.504366, -0.412676	7.50	6/2	Sand Silt	6.0x10${^6}$	1.3x10${^6}$	4.2x10${^6}$
GRA-05	39.504205, -0.412389	7.31	5/4	Sand Silt	1.0x10${^7}$	5.1x10${^5}$	3.3x10${^6}$
GRA-06	39.504103, -0.412168	7.05	7/2	Sandy	5.4x10${^7}$	1.0x10${^6}$	4.6x10${^6}$
GRA-07	39.504459, -0.411671	6.86	6/2	Sandy	8.5x10${^7}$	3.2x10${^6}$	3.1x10${^6}$
GRA-08	39.504941, -0.411895	6.35	6/6	Sandy	1.2x10${^7}$	2.5x10${^6}$	5.5x10${^5}$
GRA-09	39.505242, -0.412607	6.36	7/2	Sandy	6.0x10${^7}$	1.8x10${^6}$	1.4x10${^7}$
GRA-10	39.505255, -0.413009	7.41	3/2	Loamy Sandy	6.5x10${^7}$	8.2x10${^4}$	4.2x10${^6}$
GRA-11	39.505336, -0.413664	7.60	4/1	Sandy	3.3x10${^7}$	1.2x10${^6}$	8.4x10${^5}$
GRA-12	39.506009, -0.412904	6.47	5/3	Sandy	5.8x10${^7}$	1.9x10${^6}$	9.2x10${^6}$
GRA-13	39.505909, -0.412259	7.53	4/4	Loamy Sandy	8.0x10${^7}$	1.8x10${^6}$	2.4x10${^6}$
GRA-14	39.506547, -0.412587	7.02	7/6	Sandy	4.5x10${^6}$	3.3x10${^5}$	5.7x10${^5}$
GRA-15	39.506947, -0.412266	7.00	4/4	Loamy Sandy	1.7x10${^7}$	1.7x10${^5}$	3.9x10${^6}$
GRA-16	39.507239, -0.412076	7.27	2/2	Loamy Sandy	2.0x10${^6}$	1.3x10${^4}$	2.1x10${^5}$
GRA-17	39.506842, -0.411706	6.70	6/2	Sandy	1.6x10${^7}$	4.4x10${^5}$	2.8x10${^5}$
GRA-18	39.506968, -0.411554	6.70	5/3	Sandy	1.1x10${^7}$	4.8x10${^6}$	4.3x10${^6}$
GRA-19	39.506331, -0.411909	6.65	6/1	Sandy	5.5x10${^7}$	1.5x10${^6}$	9.7x10${^6}$
GRA-20	39.505634, -0.411617	7.03	6/3	Sandy	1.1x10${^7}$	1.4x10${^6}$	8.4x10${^5}$
GRA-21	39.505195, -0.411243	6.02	6/4	Sandy	7.9x10${^7}$	1.3x10${^7}$	9.0x10${^6}$
GRA-22	39.504981, -0.411605	6.77	7/2	Sandy	9.0x10${^6}$	3.0x10${^3}$	4.3x10${^5}$
GRA-23	39.505228, -0.412055	6.06	6/3	Sandy	1.7x10${^7}$	3.6x10${^6}$	6.4x10${^6}$
GRA-24	39.504950, -0.412158	6.19	5/4	Sandy	3.4x10${^7}$	2.1x10${^6}$	1.1x10${^6}$
GRA-25	39.505101, -0.412595	6.39	6/6	Sandy	5.8x10${^6}$	6.4x10${^5}$	7.6x10${^5}$

**Fig. 2:**
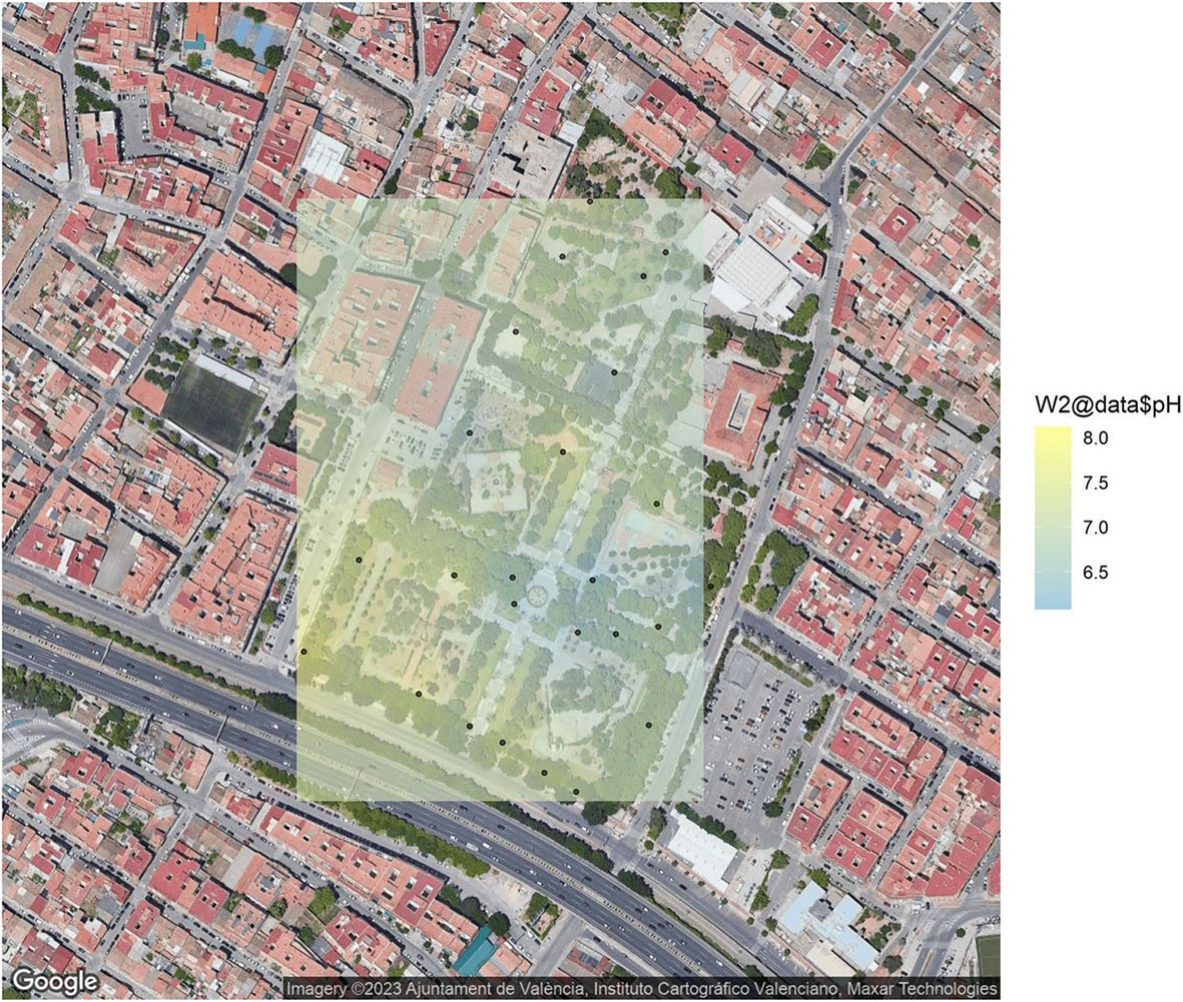
Kriging graphic of the pH measurement at the Park of La Granja (Google Maps)

The microbial quantities counted in this park were between 2.0x10${^6}$ to 8.0x10${^7}$ ufc$\cdot$g$^{-1}$. In a parallel culture test in MA we have determined the concentration of fungi. The percentage of fungal species in sandy soils range from 0 to 44% of the colonies found, while the quantities of these eukaryotes is generally lower (1-9 %) in sand silt soils. A high number of Enterobacteriaceae was also found, ranging from 4.3x10${^5}$ to 1.4x10${^7}$ ufc$\cdot$g$^{-1}$. A more detailed analysis of these groups is described later. The values of pH were also variable, as soils of pH 6.02 to 8.14 were measured, from moderately acidic to moderately alkaline according to the Natural Resources Conservation Service classification [29]. To evaluate these results as a whole we have performed a Kriging analysis, including these results. We have found that boundaries of the park, exhibit higher values of pH in comparison with inner areas (Fig. 2).

Moreover, according to the results observed in Table 2, there is an inverse linear correlation, at 95% significance, between pH values and the number of enterobacteria and fungi reported, which means that for acid soils (with low pH values) these microorganisms can grow easily (as expected).

pH is known to be a critical factor for shaping the biogeographical microbial patterns as reported in previous studies on the bacterial diversity [30]. As expected pH played a definite role on the diversities and compositions of bacterial community [31, 32]. Moreover, in accord with the above mentioned results concerning the geochemical property of soils, the number of heterotrophic total bacteria/fungi was similar at several sampling points 1.

**Table 2:** Microorganisms and pH. Correlation with measured values

	Total (ufc/g)	Fungi (ufc/g)	Enterobacteria (ufc/g)	pH	Color
Total (ufc/g)	1.00	0.42$^{*}$	0.56$^{*}$	-0.19	0.05
Fungi (ufc/g)	0.42$^{*}$	1.00	0.42$^{*}$	-0.51$^{*}$	0.20
Enterobacteria (ufc/g)	0.56$^{*}$	0.42$^{*}$	1.00	-0.42$^{*}$	0.18
pH	-0.19	-0.51$^{*}$	-0.42$^{*}$	1.00	-0.56$^{*}$
Color	0.05	0.20	0.18	-0.56$^{*}$	1.00

N=25; *: p<0.05; **: p<0.001

**Fig. 3:**
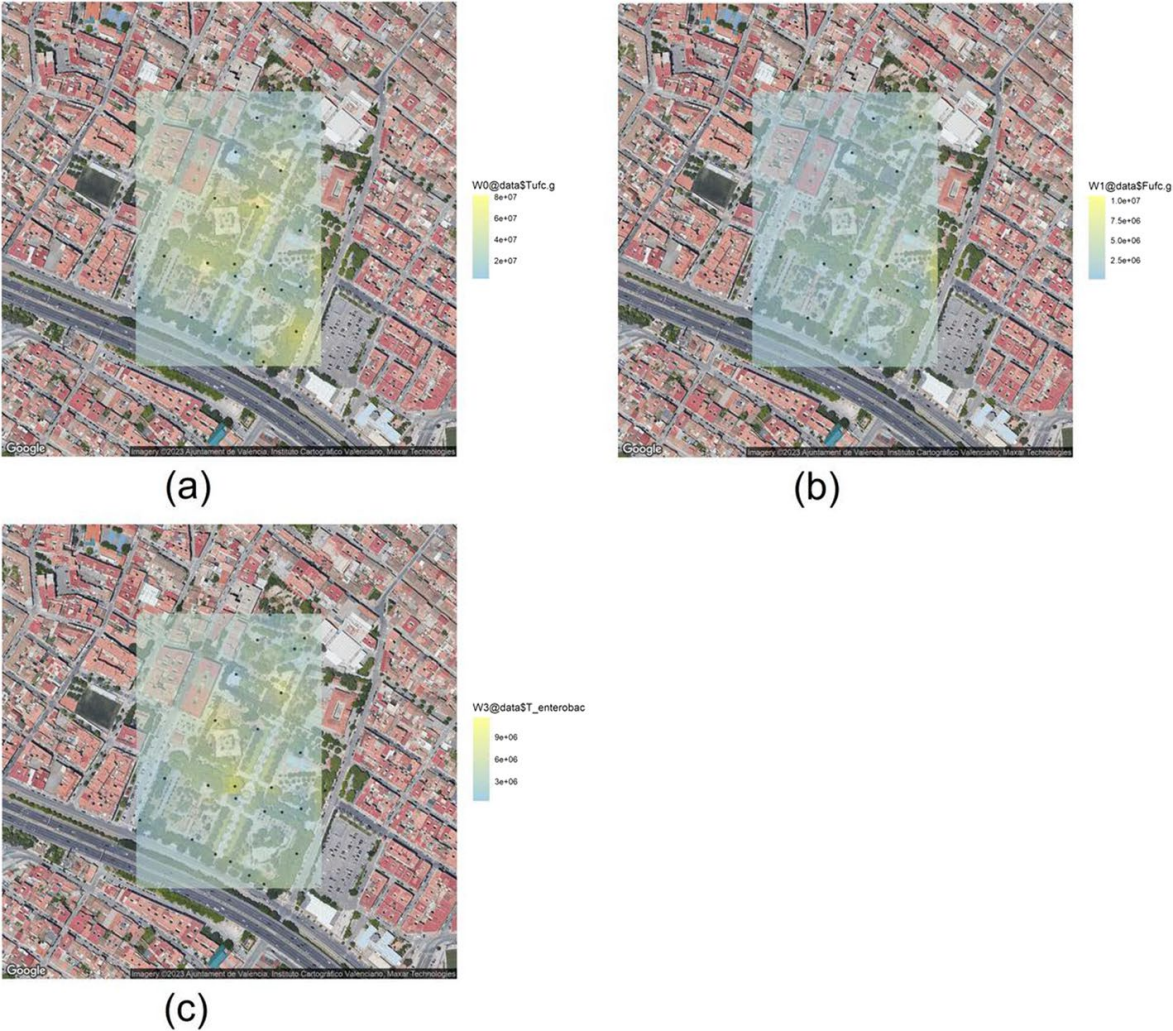
Kriging plots at the Park of La Granja (Google Maps): **a** Total microorganisms (ufc$\cdot$g$^{-1}$), **b** Fungi (ufc$\cdot$g$^{-1}$) and **c** Enterobacteria (ufc$\cdot$g$^{-1}$)

**Fig. 4:**
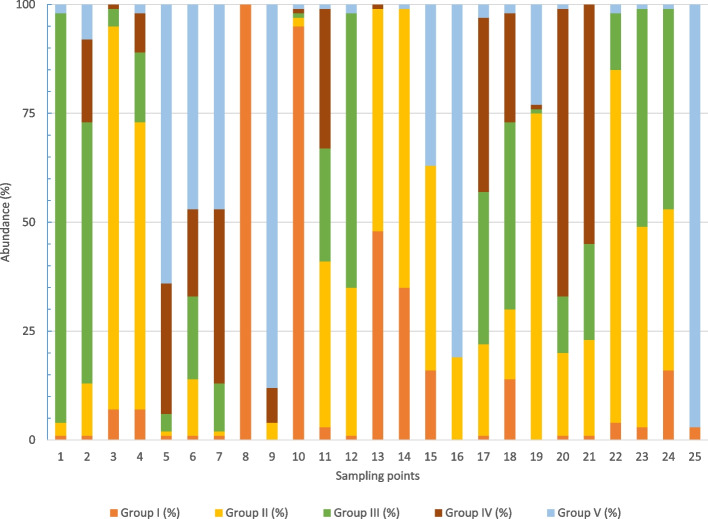
Percentage of enterobacterial isolates at the Park of La Granja: **a** G.I, **b** G.II, **c** G.III, **d** G.IV and **e** G.V

The spatial distribution of different groups of microorganisms inside the study area is shown in (Fig. 3). Although there is a variable distribution of microorganisms there is a positive correlation between the total numbers and the quantities of enterobacteriaceae. Moreover, lower values of pH were also correlated with higher quantities of fungus. In the sampling point 9 soil sample, numbers of enterobacteria were more than ten times greater than that at most of the other sampling points. The detection of Enterobacteriaceae in soils is usually an indication of the deposition of fecal remains on them. To evaluate the concentrations of Enterobacteriaceae, we spread a dilution of the samples in MKA [33]. MKA is a selective and differential culture medium for bacteria designed to selectively isolate Gram-negative and enteric bacilli. It contains bile salts (to inhibit most Gram-positive bacteria), crystal violet dye (to inhibit some Gram-positive bacteria), neutral red dye (which turns pink if the isolates are able to ferment lactose). In the basis of these facts, we have grouped the different isolates. Group I (Lac positive), II (Lac negative), III (slow), IV (mucoid colonies) and V (other) (Fig. 4).

**Fig. 5:**
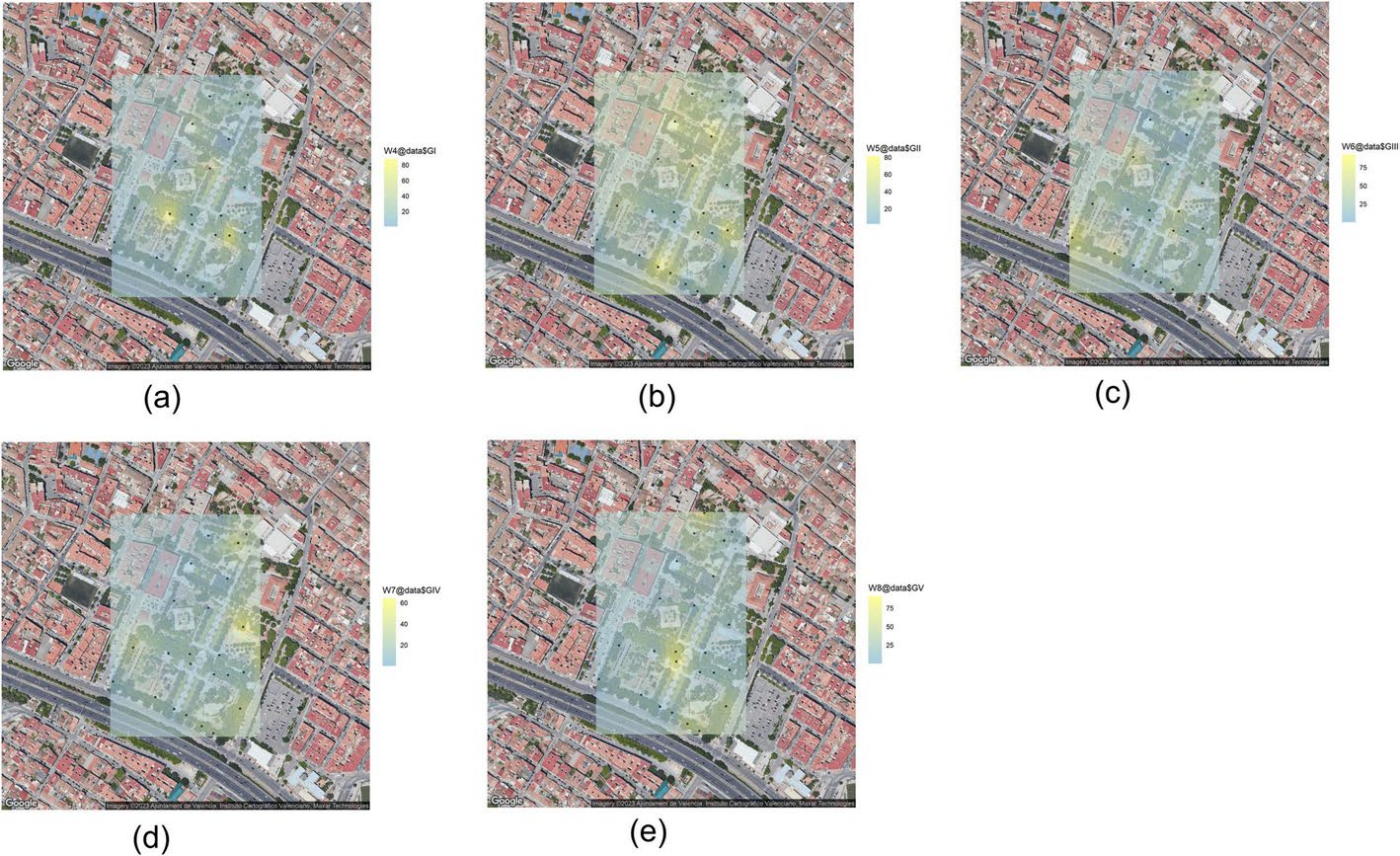
Kriging plots of percentage of enterobacterial isolates at the Park of La Granja (Google Maps): **a** G.I, **b** G.II, **c** G.III, **d** G.IV and **e** G.V

There is a high degree of variability regarding the distribution of the Enterobacteriaceae among the five subgroups. In some points, as GRA-01 or GRA-25 one of them predominates over the others, with almost a single type of colonial morphology being observed among the different colonies observed on the plates. At other sampling points, the variability is much greater, since there is a colonial distribution that allows us to assign colonies to the five previously defined subgroups. This observation can be corroborated by evaluating the results of the Kriging analysis carried out for each of the five categories described (Fig. 5). Thus, we observe that subgroups I and V (Fig. 5a and e) are distributed in specific focus in the inner part of the park. For its part, subgroup II (Fig. 5b) presents a more spread distribution throughout the whole park. Finally, subgroup III and IV (Fig. 5c and d) show a focused distribution in specific areas of the outer part of the park.

**Table 3:** Partial characterization and identification of selected isolates

Code	16S rRDNA	rDNA$^{1}$	MALDI-TOF	M-TOF$^{2}$	M-TOF$^{3}$
		score$^{1}$		score$^{2}$	C index$^{3}$
GRA-01A	*Pseudomonas koreensis*	97.44	*Pseudomonas koreensis*	2.45	A
GRA-01B	unidentified	-	*Pantoea ananatis*	2.45	A
GRA-02D	*Pseudomonas sp.*	84.06	*Pseudomonas putida*	1.71	B
GRA-03A	*Leclercia adecarboxylata*	85.59	*Leclercia adecarboxylata*	2.53	A
GRA-05A	*Salmonella sp.*	93.13	*Salmonella sp.*	1.49	C
GRA-05B	*Escherichia coli*	74.09	*Pantoea calida*	1.72	B
GRA-06E	*Pseudomonas thivervalensis*	85.19	*Pseudomonas brassicacearum*	2.16	A
GRA-07D	unidentified	-	*Aeromonas molluscorum*	1.44	C
GRA-11C	unidentified	-	*Serratia fonticola*	1.51	C
GRA-12A	unidentified	-	*Klebsiella aerogenes*	2.42	A

$^{1}$ rDNA 16S sequencing significance

$^{2}$ MALDI-TOF *Score significance*: <1.69, no identification; 1.70-1.99, low confidence; 2.00-3.00, high confidence

$^{3}$ MALDI-TOF *Consistency index*: A, high; B, low; C, no consistency

### Identification and partial characterization of selected Enterobacteriaceae isolates

A total of 10 morphological and physiologically different bacteria isolated from MKA plates were selected for further analysis (Table 3). 16S rDNA sequencing and MALDI-TOF-MS was carried out.

All the selected colonies could be identified by MALDI-TOF. Half of them (five), with a high degree of reliability (A), two with intermediate reliability (B) and three with low reliability (C). In some cases, the identification could also be corroborated by 16S rDNA sequencing. The common use of both techniques allowed us to identify the ten isolates at least at the gender level. Some soil microorganisms, such as *Pantoea ananatis*, are considered as plant pathogens but some strains are useful from a biotechnological point of view, and they are used for plant growth promotion [34]. Soil can also be a good place to discover new commercial interesting strains of microorganisms. *Pseudomonas koreensis* is a putative producer of extracellular lipases [35].

### Lipases screening

In a prospective trial, 20 colonies isolated from each of the 25  sampling points (a total of 500 colonies) were assayed for lipase action in an artificial medium (Tween-80 agar medium). A total of 17 different isolates were found to be lipase producing microorganisms, four of them exhibiting strong precipitation halos higher than 1 cm on the plates. These strains were selected for 16S rDNA identification (Table 4).

**Table 4:** Semiquantitative lipase determination

Sample code	Isolate number	Activity	16S rDNA identification	Percent identity %
GRA-01	01	+		
GRA-02	01	+		
GRA-04	01	++	*Pseudomonas sp*	88.00
GRA-04	02	+		
GRA-05	01	+		
GRA-06	03	+		
GRA-07	02	++	*Acinetobacter vivianii*	84.23
GRA-08	02	+		
GRA-10	02	++	*Pseudomonas sp*	81.20
GRA-13	03	+		
GRA-15	01	+		
GRA-15	04	++	*Streptomyces sp*	92.26
GRA-17	01	+		
GRA-17	02	+		
GRA-17	03	+		
GRA-17	05	+		
GRA-23	03	+		

$^{+}$ Medium activity (slight precipitation);

$^{++}$ High activity (strong precipitation)

Two of the isolates were identified as *Pseudomonas sp*, and the others were *Streptomyces sp* and *Acinetobacter vivianii*. Lipases (glycerol ester hydrolases; EC 3.1.1.3) are important enzymes which, due to their ability to catalyze a number of reactions interesting for both academia and industry. By the other side, there is known to be a protective action of soil-inhabiting organisms.

The isolation of these microorganisms in soil can indicate a contaminated area. The existence of an adaptive capability of soil bacteria to the effects of environmental factors as high concentration of oil products may be a warning of ecological malfunction of soil as “bacterial filter” and can be a threat for humans. The bacterial genus *Pseudomonas* is a prolific producer of a number of extracellular enzymes including lipase [36]. *Acinetobacter vivianii* and *Streptomyces sp* isolated from soils have also previously been reported as exocellular enzyme producers [37, 38]. Their isolation can be correlated with oily wastes (Table 5).

**Table 5:** Correlation with Kriging values

	Tufc.g	Fungi_ufc.g	pH	T_enterobac	GI	GII	GIII	GIV	GV
Tufc.g	1.00	0.31$$^{**}$$	-0.24$$^{**}$$	0.54$$^{**}$$	0.26$$^{**}$$	-0.15$$^{**}$$	-0.20$$^{**}$$	0.05	0.10$$^{*}$$
Fungi_ufc.g	0.31$$^{**}$$	1.00	-0.56$$^{**}$$	0.23$$^{**}$$	-0.10$$^{*}$$	-0.04	-0.05	0.47$$^{**}$$	-0.16$$^{**}$$
pH	-0.24$$^{**}$$	-0.56$$^{**}$$	1.00	-0.45$$^{**}$$	-0.10$$^{*}$$	-0.12$$^{**}$$	0.41$$^{**}$$	-0.02	-0.20$$^{**}$$
T_enterobac	0.54$$^{**}$$	0.23$$^{**}$$	-0.45$$^{**}$$	1.00	-0.02	0.10$$^{*}$$	-0.16$$^{**}$$	-0.24$$^{**}$$	0.25$$^{**}$$
GI	0.26$$^{**}$$	-0.10	-0.10$$^{**}$$	-0.02$$^{*}$$	1.00	-0.06	-0.32$$^{**}$$	-0.39$$^{**}$$	-0.17$$^{**}$$
GII	-0.15$$^{**}$$	-0.04	-0.12$$^{**}$$	0.10$$^{*}$$	-0.06	1.00	-0.33$$^{**}$$	-0.31$$^{**}$$	-0.36$$^{**}$$
GIII	-0.20$$^{**}$$	-0.05	0.41$$^{**}$$	-0.16$$^{**}$$	-0.32$$^{**}$$	-0.33$$^{**}$$	1.00	0.00	-0.43$$^{**}$$
GIV	0.05	0.47$$^{**}$$	-0.02	-0.24$$^{**}$$	-0.39$$^{**}$$	-0.31$$^{**}$$	0.00	1.00	-0.10$$^{*}$$
GV	0.10$$^{*}$$	-0.16$$^{**}$$	-0.20$$^{**}$$	0.25$$^{**}$$	-0.17$$^{**}$$	-0.36$$^{**}$$	-0.43$$^{**}$$	-0.10$$^{*}$$	1.00

N=896; *: p<0.05; **: p<0.001

In this study, shown in Table 5, we are interested in the effect of pH in soils (as an objective physic-chemical parameter) in the growth of different microorganisms. The analysis of 25 samples allowed to find inverse correlation of pH with the number of enterobacteria and inverse correlation with the number of fungi. As a direct consequence, we can infer that in alkaline soils, the conditions to grow of these microorganisms are tougher and harder.

With the Kriging technique (and spatial resolution of 0.0001 in the GPS coordinates of the grid used), we obtained 896 samples. This fact allows us to obtain better significance with the cross-correlation studies and also a better adjustment of the correlation values. Here, we find significant inverse/negative correlation of pH with the total number of isolates (Total m.o.), with the number of fungi and with the number of enterobacteria. Something similar happens with the different groups considered, from I to V except for group IV (mucoid colonies) which is not significant, and group III (slow) which offers a direct correlation with soils’ pH.

## Conclusions

Urban parks are a fundamental part of our cities. Performing soil scrutiny studies allows determining which microbial communities inhabit it. The performance of relatively simple sequencing analyzes (16S rDNA), in combination with more sophisticated techniques such as MALDI-TOF, allows a global characterization of these communities. Carrying out physical-chemical measurements (determination of pH and color of the soils), and a combined study of all these variables using computer techniques (Kriging) provides a global vision of the situation of an environment such as the one chosen for this study. Increased use of public spaces by humans is linked to increased antibiotic concentrations in a given ecosystem such as a park, and can influence both antibiotic resistance and microbial population dynamics [27]. Understanding the evolution of microbial populations, especially in times of increasing resistance, is of interest. It is still unknown how these different environmental and individual determinants are distributed in time and space, and their potential influences on the emergence of resistance. Future contributions from our group and others will contribute to a better understanding of the overall process, in order to certify the healthiness of a public space. Knowing the microbial variety in a public space makes it possible to determine the potential health risks that the appearance of undesirable microorganisms could have, either because of their intrinsic characteristics or because of their concentration.

## Supplementary Information

**Additional file 1.**

## References

[1] Ministerio de Fomento. Atlas Digital de las Áreas Urbanas. http://atlasau.fomento.gob.es/ayuda/inicio.htm. Accessed 03 Jan 2021.

[2] Yuan DG, Zhang GL, Gong ZT (2008). Numerical Approaches to Identification of Characteristic Soil Layers in an Urban Environment. Project supported by the National Natural Science Foundation of China (No. 40625001) and the Knowledge Innovation Program of the Chinese Academy of Sciences (No. KZCX2-YW-409). Pedosphere.

[3] Moore M, Gould P, Keary BS (2003). Global urbanization and impact on health. Int J Hyg Environ Health..

[4] Cohen B (2006). Urbanization in developing countries: Current trends, future projections, and key challenges for sustainability. Technol Soc..

[5] Ferreira CSS, Walsh RPD, Ferreira AJD (2018). Degradation in urban areas. Curr Opin Environ Sci Health..

[6] Qadir M, Wichelns D, Raschid-Sally L, McCornick PG, Drechsel P, Bahri A (2010). The challenges of wastewater irrigation in developing countries. Agric Water Manag..

[7] Bastida F, Moreno JL, García C, Hernández T (2007). Addition of urban waste to semiarid degraded soil: Long-term effect. Pedosphere..

[8] Pavao-Zuckerman MA (2008). The nature of urban soils and their role in ecological restoration in cities. Restor Ecol..

[9] Mesjasz-Lech A (2014). Municipal waste management in context of sustainable urban development. Procedia-Soc Behav Sci..

[10] Santamaría J, Toranzos GA (2003). Enteric pathogens and soil: A short review. Int Microbiol..

[11] Perera LN, Mafiz AI, Amarasekara NR, Chang E, Rao VBK, Zhang Y (2020). Antimicrobial-resistant E. coli and Enterococcus spp. Recovered from urban community gardens. Food Control.

[12] Yuan W, Zhang Y, Riaz L, Yang Q, Du B, Wang R. Multiple antibiotic resistance and DNA methylation in Enterobacteriaceae isolates from different environments. J Hazard Mater. 2021;402. 10.1016/j.jhazmat.2020.123822. Cited By 2. 10.1016/j.jhazmat.2020.123822 33254807

[13] Matthiessen L, Bergström R, Dustdar S, Meulien P, Draghia-Akli R. Increased momentum in antimicrobial resistance research. Lancet. 2016;388(10047):865. 10.1016/S0140-6736(16)31425-8. Cited By 11. 10.1016/S0140-6736(16)31425-8 27597459

[14] Mao D, Yu S, Rysz M, Luo Y, Yang F, Li F, et al. Prevalence and proliferation of antibiotic resistance genes in two municipal wastewater treatment plants. Water Res. 2015;85:458–466. 10.1016/j.watres.2015.09.010. Cited By 234. 10.1016/j.watres.2015.09.010 26372743

[15] Rodriguez-Mozaz S, Chamorro S, Marti E, Huerta B, Gros M, Sànchez-Melsió A, et al. Occurrence of antibiotics and antibiotic resistance genes in hospital and urban wastewaters and their impact on the receiving river. Water Res. 2015;69:234–242. 10.1016/j.watres.2014.11.021. Cited By 598. 10.1016/j.watres.2014.11.021 25482914

[16] Yuan W, Tian T, Yang Q, Riaz L. Transfer potentials of antibiotic resistance genes in Escherichia spp. strains from different sources. Chemosphere. 2019;246:1–9. Cited By 1. 10.1016/j.chemosphere.2019.125736 31896018

[17] Raja S, Cheema HMN, Babar S, Khan AA, Murtaza G, Aslam U (2015). Socio-economic background of wastewater irrigation and bioaccumulation of heavy metals in crops and vegetables. Agric Water Manag..

[18] Rivera-Jaimes JA, Postigo C, Melgoza-Alemán RM, Aceña J, Barceló D, López de Alda M (2018). Study of pharmaceuticals in surface and wastewater from Cuernavaca, Morelos, Mexico: Occurrence and environmental risk assessment. Sci Total Environ.

[19] Thompson JA, Pollio AR, Turk PJ. Comparison of Munsell Soil Color Charts and the GLOBE Soil Color Book. Soil Sci Soc Am J. 2013;77(6):2089–2093. 10.2136/sssaj2013.03.0117n. https://acsess.onlinelibrary.wiley.com/doi/abs/10.2136/sssaj2013.03.0117n.

[20] O’Leary WM (1989). Practical handbook of microbiology.

[21] Ahmed O, Dablool A (2017). Quality Improvement of the DNA extracted by boiling method in Gram negative bacteria. Int J Bioassays.

[22] Maicas S, Segura-Garcia J. Spatial distribution of antibiotic-producing bacteria in urban areas. A case study in Valencia (Spain). IEEE/ACM Trans Comput Biol Bioinforma. 2020;PP. 10.1109/tcbb.2020.3046557. 10.1109/TCBB.2020.3046557 33351763

[23] BLAST software. https://blast.ncbi.nlm.nih.gov/Blast.cgi. Accessed 03 Jan 2021.

[24] Maier T, Klepel S, Renner U, Kostrzewa M (2006). Fast and reliable MALDI-TOF MS-based microorganism identification. Nat Methods..

[25] Isaaks EH, Srivastava RM (1989). An Introduction to Applied Geostatistics.

[26] Cressie N (1993). Statistics for Spatial Data.

[27] RStudio. https://www.rstudio.com/. Accessed 1 Oct 2022.

[28] Lindbo DL, Kozlowski DA, Robinson C (2012). Know Soil Know Life: Physical Properties of Soil and Soil Formation.

[29] Natural Resources Conservation Service Soils, USDA. https://www.nrcs.usda.gov/wps/portal/nrcs/main/soils/survey/class/. Accessed 01 Oct 2022.

[30] Cho S, Kim M, Lee Y. Effect of pH on soil bacterial diversity. J Ecol Environ. 2016;40. 10.1186/s41610-016-0004-1.

[31] Lauber CL, Hamady M, Knight R, Fierer N (2009). Pyrosequencing-based assessment of soil pH as a predictor of soil bacterial community structure at the continental scale. Appl Environ Microbiol..

[32] Fierer N, Jackson RB (2006). The diversity and biogeography of soil bacterial communities. Proc Natl Acad Sci U S A..

[33] MacConkey AT (1908). Bile Salt Media and their advantages in some Bacteriological Examinations. J Hyg..

[34] Megías E, Junior FBR, Ribeiro RA, Ollero FJ, Megías M, Hungria M (2018). Draft genome sequence of Pantoea ananatis strain 1.38, a bacterium isolated from the rhizosphere of Oryza sativa var. Puntal that shows biotechnological potential as an inoculant. Genome Announc.

[35] Anbu P (2014). Characterization of an extracellular lipase by pseudomonas koreensis BK-L07 isolated from soil. Prep Biochem Biotechnol..

[36] Gilbert E (1993). Pseudomonas lipases: biochemical properties and molecular cloning. Enzym Microb Technol..

[37] Nemec A, Radolfova-Krizova L, Maixnerova M, Vrestiakova E, Jezek P, Sedo O (2016). Taxonomy of haemolytic and/or proteolytic strains of the genus Acinetobacter with the proposal of Acinetobacter courvalinii sp. nov. (genomic species 14 sensu Bouvet & Jeanjean), Acinetobacter dispersus sp. nov. (genomic species 17), Acinetobacter modestus sp. nov., Acinetobacter proteolyticus sp. nov. and Acinetobacter vivianii sp. nov. Int J Syst Evol Microbiol.

[38] Boran R, Ugur A, Sarac N, O C. Characterisation of Streptomyces violascens OC125-8 lipase for oily wastewater treatment. 3 Biotech. 2019;9(1). 10.1007/s13205-018-1539-x. 10.1007/s13205-018-1539-x PMC6312823 30622843

